# A surgical polypragmasy: Koninckx PR, Corona R, Timmerman D, Verguts J, Adamyan L. Peritoneal full-conditioning reduces postoperative adhesions and pain: a randomised controlled trial in deep endometriosis surgery. J Ovarian Res. 2013 Dec 11;6(1):90

**DOI:** 10.1186/1757-2215-7-29

**Published:** 2014-03-10

**Authors:** Ospan A Mynbaev, Peter Biro, Marina Yu Eliseeva, Andrea Tinelli, Antonio Malvasi, Ioannis P Kosmas, Mykhailo V Medvediev, Tatiana I Babenko, Madina I Mazitova, Sergei S Simakov, Michael Stark

**Affiliations:** 1 The International Translational Medicine & Biomodeling Research Group, Department of Applied Mathematics, Moscow Institute of Physics & Technology (State University), 9 institutsky per, Dolgoprudny, Moscow Region 141700, Russia; 2 The Institute of Anaesthesiology, University Hospital, 100 Rämistrasse, Zürich CH-8091, Switzerland; 3 The Department of Obstetrics, Gynecology & Reproductive Medicine, Peoples’ Friendship University of Russia, 21/3 Miklukho-Maklay str, Moscow 117198, Russia; 4 The Department of Obstetrics and Gynaecology, Division of Experimental Endoscopic Surgery, Imaging, Minimally Invasive Therapy and Technology, Vito Fazzi Hospital, Piazza Muratore, Lecce 73100, Italy; 5 Department of Obstetrics and Gynecology, Santa Maria Hospital, 314/A Via Alcide De Gasperi, Bari 70125, Italy; 6 The Department of Obstetrics & Gynecology, Xatzikosta General Hospital, Avenue General Makriyannis, Ioannina 45001, Greece; 7 The Department of Obstetrics & Gynecology, State Establishment “Dnepropetrovsk Medical Academy of Health Ministry of Ukraine”, 9 Dzerzhinky str, Dnepropetrovsk 49044, Ukraine; 8 The Department of Obstetrics & Gynecology, Stavropol State Medical University, 310 Mira str, Stavropol 355017, Russia; 9 The Department of Obstetrics and Gynecology, Kazan State Medical Academy, 11/1 Mushtary, Kazan 420012, Russia; 10 The New European Surgical Academy, 21 Unter den Linden, Berlin 10117, Germany

## 

Dear Sir,

In clinical trials most adhesion prevention methods fail. Therefore we have read the article by Koninckx et al. [1] with great interest. They aim “… to perform a translational proof of concept trial to investigate the effect of full-conditioning (FC) in the human upon CO_2_ resorption during surgery, …”.

Congratulations to the authors are in order for their valuable clinical results which have demonstrated the efficiency of their attempts to prevent postsurgical adhesions, reducing pain and modulated posttraumatic inflammation with lower postoperative C-reactive protein (CRP) values and accelerated recovery. Since huge obstacles in the setup of clinical studies are met when second-look laparoscopy (SLL) is required, most studies evaluate the efficiency of adhesion prevention adjuvants on the basis of experiments only. It is indeed difficult to organize such kind of clinical trials due to both severe ethical and financial issues and patient requirement difficulties. Therefore, clinical trials aimed to evaluate postsurgical adhesion formation by SLL should be based on a well-defined study question, a distinct design and strictly specified patient selection, all of which may lead to evidence-based conclusions, albeit in a limited number of homogenous population.

However, some shortcomings of this study [1], which might lead to misleading conclusions, and the authors’ disregard of well-known undesirable N_2_O side effects have coerced us to write this letter.

Professor Koninckx is an outstanding, worldwide authority in this field who performs high-quality surgical treatment of deep endometriosis. Therefore we may safely assume that for deep endometriosis excision in both groups, identical surgical procedures were performed and that presurgical randomization and postsurgical follow-up were also done up to standard.

However, the patients in the FC group experienced surgical polypragmasy, namely: 1) a humidified pneumoperitoneum gas mixture (86% CO_2_ + 10% N_2_O + 4% O_2_) with controlled gas temperature (31°C); 2) peritoneal cooling (up to 30°C) by sprinkling 2–3 ml/min of Ringers lactate with heparin (1000 IU/L) at room temperature; 3) a Hyalobarrier gel (HBG) application, and 4) 5 mg of dexamethasone administered intramuscularly at the end of surgery. The women in the control group however, were operated by standard laparoscopy with humidified CO_2_ only and these two completely different treatment approaches were compared. Subsequently, according to the authors’ conclusions, successful adhesion prevention treatment was estimated by FC only.

We quote: “This translational research confirms in the human the efficacy of FC in reducing CO_2_ resorption and adhesions with in addition less postoperative pain, lower postoperative CRP concentrations and an accelerated recovery” [1].

It is difficult to identify the cause of the beneficial adhesion prevention impact because of the excessive combination of several factors. Indeed, it is difficult to accept the authors’ conclusion that the adhesion prevention effect is related with FC only, since in these patients HBG was also applied and dexamethasone was administered. Therefore, the abstract section does not reflect their results at all and may lead JOR readers astray.

Another limitation of this study lies in the small number of patients and the complexity of the adhesion score. In the compared groups it is difficult to distinguish between size and severity of adhesions separately. Moreover, upon initial surgery, most patients had already had adhesions resulting from previous surgery. It is a well-known fact that the adhesion score analysis after adhesiolysis is different from that of adhesions after first surgery. It is quite clear that all of these factors, combined with the population heterogeneity in these groups which only have a small number of patients, may lead to biased results.

We previously demonstrated that reduced blood gas changes during CO_2_ pneumoperitoneum are associated with mixed gas insufflation (MGI) since even a small concentration of O_2_ added to CO_2_ results in lower end tidal CO_2_ (P_ET_CO_2_) values and slight changes in blood gas parameters in comparison with those of pure CO_2_ insufflation in rabbits [2,3]. Also, MGI has a significant impact on ventilation parameters [4]. Subsequently, it is obvious that during surgery, patients in the FC group require less increased tidal volume (TV) and lower ventilation frequency than patients in the standard pneumoperitoneum group. However, this conclusion should be drawn based upon a comprehensive analysis of anesthesiological management including full respiratory and blood gas, acid base parameters without combining raw data of P_ET_CO_2,_ TV and frequency of ventilation.

In comparison with the traditional mechanism of adhesions (Figure 1A), N_2_O impact on adhesion formation may be related with the activation of several N_2_O-induced pathways (Figure 1B) with rise of Hcys content [5] and an increased cysteine concentration [6], which then possibly results in weakened collagen cross-linking in the collagen network between fibroblasts [7]; N_2_O induced-oxidative stress and DNA damage [8]; apoptosis by caspase-3 activation [9] in adhesion fibroblasts with increased genome instability.

**Figure 1:**
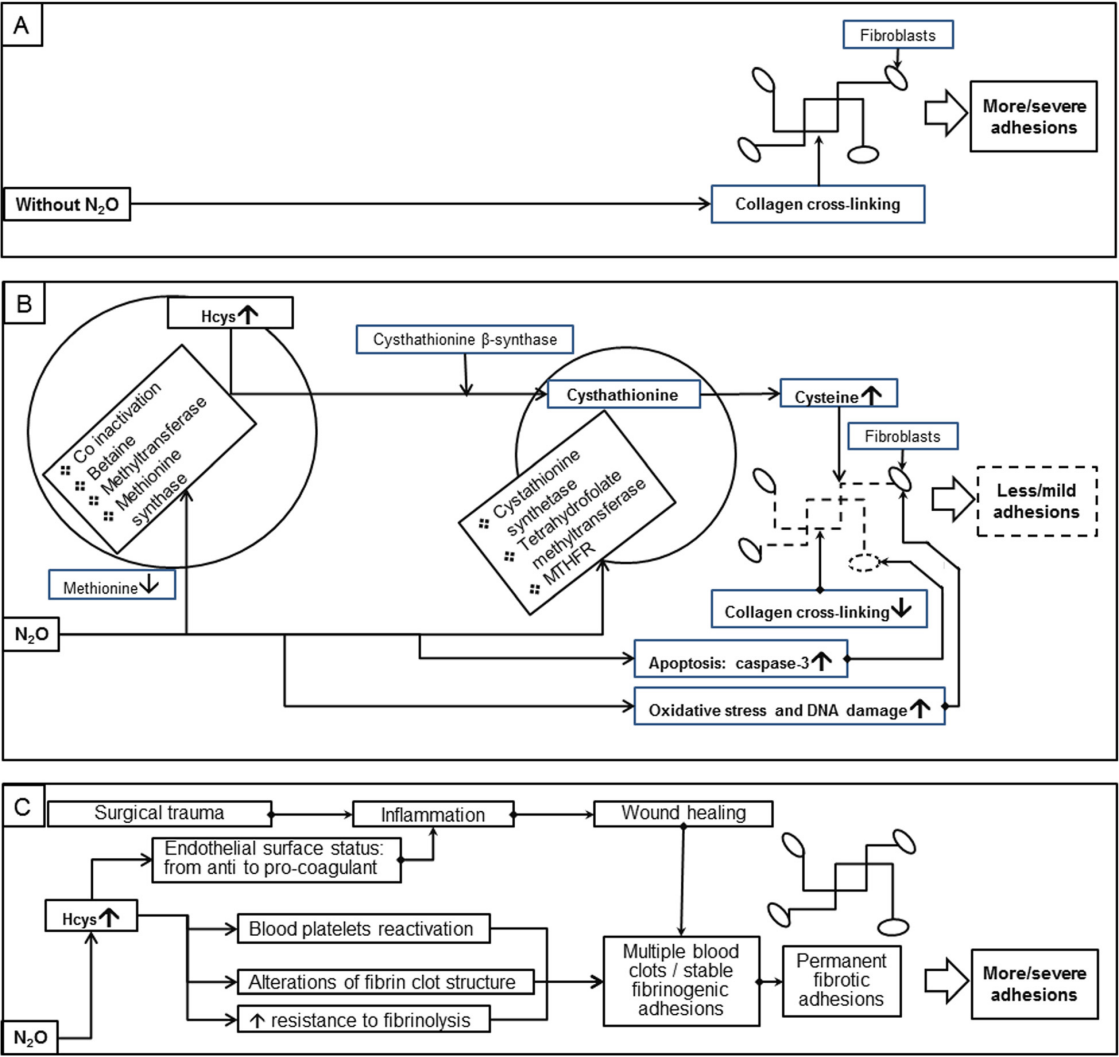
**Disputable mechanisms of the addition of N**_**2**_**O to the CO**_**2 **_**pneumoperitoneum impact on adhesion formation. A)** Traditional mechanism of multiple severe adhesion formation, which is not related with N_2_O. **B)** The antiadhesiogenic impact of N_2_O. Metabolic pathways affected by N_2_O included interactions with the cobalt atom (Co), betaine, methyltransferase, methionine synthase with increased homocysteine (Hcys) and decreased methionine [5,6]. Then, several enzyme deficiencies can also be a pathway for decreased postsurgical adhesions such as acystathionine synthetase, tetrahydrofolate methyltransferase and 5,10-methylenetetrahydrofolate reductase (MTHFR), which catalyze the synthesis of cystathionine from Hcys [5,6] with subsequent increased cysteine concentration, which then results in weakened collagen cross-linking [7] in the collagen network between fibroblasts; N_2_O induced-oxidative stress, DNA damage [8] and apoptosis by caspase-3 activation [9] in these virgin adhesion fibroblasts with increased genome instability. **C)** A possible mechanism of the adhesiogenic impact of N_2_O: The following changes are associated with increased Hcys concentration: shift of vascular endothelium surface from anti- to pro-coagulant status; reactivation of blood platelets; alterations of several intrinsic and extrinsic targets in the coagulation and fibrinolysis system with modification of the blood clot/fibrin clot structure; increased resistance of fibrin clot to fibrinolysis [13] with increased adhesion formation potential.

The authors stated that N_2_O is safer than CO_2_ due to its high solubility in water and the higher lung exchange capacity [1]. However, according to available literature, the administration of N_2_O is associated with neuro-apoptotic/neurotoxic [5,6] genotoxic effects [8] and changes in cobalamin (Vitamin B12), methionine synthase and Hcys metabolism [5,6] the latter being an α-amino acid biosynthesized from methionine by catabolism.

Through these signaling pathways an increased concentration of blood N_2_O [5,6] is associated with the expansion of air-filled spaces such as bowel, and pneumothorax [10,11]; a dose-dependent depression of the ventilatory response to hypoxemia [12]; increased DNA damage [8]; endothelial dysfunction with elevated procoagulant status [13] with increased risk of thromboembolism, atherosclerosis and cardiovascular diseases [5,6,13,14] and long term risk of myocardial infarctions [15].

It can be put forward that multiple blood clots in the lesion sites will be stabilized as long-term fibrinogenic adhesions transforming later to fibrotic adhesions (Figure 1C) due to changes of the vascular endothelium surface from an anti- to a procoagulant status, the reactivation of blood platelets involving several intrinsic and extrinsic targets in coagulation and fibrinolysis, resulting in alterations of blood clot/fibrin clot structure, and its increased resistance to fibrinolysis [13].

Surprisingly, several risk factors are related with reproductive functioning, such as: a reduced fertility [16]; an elevated risk of spontaneous abortion [17]; in rats, changes in the luteinizing hormone releasing hormone [18]; N_2_O-induced teratogenicity [19], are claimed to be a result of N_2_O administration.

These N_2_O effects, combined with other factors due to polypragmasy with multiple compounds of surgical procedures, may lead to unexpected and undesirable side effects when, in various circumstances, its action pathways remain unclear.

Although the clinical relevance of the unfavorable side effects of N_2_O remains undetermined [12,20] we should be prudent in introducing N_2_O as a safe additive compound for the pneumoperitoneum. Moreover, if we take into account the metabolic, procoagulant and DNA damaging properties of N_2_O it is clear that a spectrum of beneficial and undesirable effects of N_2_O being insufflated intraperitoneally under pressure have not yet been fully appreciated.

We suggest that the influence of N_2_O as an additional component gas for the pneumoperitoneum should be investigated in different animal models evaluating a wide range of physiological parameters including blood gases, acid base balance, oxygen/oximetry and metabolite values and this in well-designed experimental studies. Special attention should be given to local intraperitoneal changes such as oxidative stress parameters, the response of peritoneal macrophages and the role of immune reaction pathways. These studies are needed to demonstrate the impact of both, CO_2_ and N_2_O on postsurgical adhesions and the homeostasis. This combination might indeed reduce or even solve the adhesion problem, but as long as experimental studies by other research groups do not prove the benefits nor examine the calculated risks, we should continue to use CO_2,_ which is a well-established gas with known pathophysiological mechanisms.

At NESA, we believe in, and are initiating evidence-based studies toward the standardization of surgical procedures in order to avoid the implementation of superfluous technologies instead of relying on surgical excellence. The concept of simple surgical procedures performed with a limited number of instruments and surgical equipment was suggested in cases where an adhesion prevention strategy should be implemented by a personalized approach taking into account individual genetic and constitutional predispositions. Any surgical tool, procedure or combination of gases should be introduced only after it has been proved to add value to existing ones; therefore we should continue to examine the effect of gas mixtures as potential agents leading to adhesion free endoscopy.

In conclusion, this FC clinical trial definitely is a step forward in the surgical treatment of severe deep endometriosis. Future studies with simplified MGI may cast further light on the mechanism of this strategy’s adhesion prevention impact since pure CO_2_ during laparoscopic surgery produces severe acidosis, blood gas and acid base changes with increased intraperitoneal insufflation pressure.

## Abbreviations

CRP: C-reactive protein; SLL: Second look laparoscopy; FC: Full-conditioning; MGI: Mixed gas insufflation; CO2: Carbon dioxide; N2O: Nitrous oxide; O2: Oxygen; HBG: Hyalobarrier gel; JOR: Journal of ovarian research; TV: Tidal volume; Hcys: Homocysteine; DNA: Deoxyribonucleic acid; NESA: New European Surgical Academy; Polypragmasy: The use of multiple therapeutic modalities to manage a single condition. (^#^Segen’s Medical Dictionary. © 2012 Farlex, Inc. All rights reserved).

## Competing interests

The authors declare that they have no competing interests.

## Authors’ contributions

In the design of this letter all authors contributed equally and approved the final manuscript.
